# NEMA NU 2–2007 performance characteristics of GE Signa integrated PET/MR for different PET isotopes

**DOI:** 10.1186/s40658-019-0247-x

**Published:** 2019-07-04

**Authors:** Paulo R. R. V. Caribé, M. Koole, Yves D’Asseler, Timothy W. Deller, K. Van Laere, S. Vandenberghe

**Affiliations:** 1 Medical Imaging and Signal Processing – MEDISIP, Ghent University, Corneel Heymanslaan 10, 9000 Ghent, Belgium; 2 Division of Nuclear Medicine and Molecular Imaging, UZ/KU Leuven, Herestraat 49 B-3000, Leuven, Belgium; 3 GE Healthcare, Waukesha, WI 53188-1678 USA

**Keywords:** PET/MR, NEMA NU 2–2007, ^18^F, ^68^Ga, ^90^Y

## Abstract

**Background:**

Fully integrated PET/MR systems are being used frequently in clinical research and routine. National Electrical Manufacturers Association (NEMA) characterization of these systems is generally done with ^18^F which is clinically the most relevant PET isotope. However, other PET isotopes, such as ^68^Ga and ^90^Y, are gaining clinical importance as they are of specific interest for oncological applications and for follow-up of ^90^Y-based radionuclide therapy. These isotopes have a complex decay scheme with a variety of prompt gammas in coincidence. ^68^Ga and ^90^Y have higher positron energy and, because of the larger positron range, there may be interference with the magnetic field of the MR compared to ^18^F. Therefore, it is relevant to determine the performance of PET/MR for these clinically relevant and commercially available isotopes.

**Methods:**

NEMA NU 2–2007 performance measurements were performed for characterizing the spatial resolution, sensitivity, image quality, and the accuracy of attenuation and scatter corrections for ^18^F, ^68^Ga, and ^90^Y. Scatter fraction and noise equivalent count rate (NECR) tests were performed using ^18^F and ^68^Ga. All phantom data were acquired on the GE Signa integrated PET/MR system, installed in UZ Leuven, Belgium.

**Results:**

^18^F, ^68^Ga, and ^90^Y NEMA performance tests resulted in substantially different system characteristics. In comparison with ^18^F, the spatial resolution is about 1 mm larger in the axial direction for ^68^Ga and no significative effect was found for ^90^Y. The impact of this lower resolution is also visible in the recovery coefficients of the smallest spheres of ^68^Ga in image quality measurements, where clearly lower values are obtained. For ^90^Y, the low number of counts leads to a large variability in the image quality measurements. The primary factor for the sensitivity change is the scale factor related to the positron emission fraction. There is also an impact on the peak NECR, which is lower for ^68^Ga than for ^18^F and appears at higher activities.

**Conclusions:**

The system performance of GE Signa integrated PET/MR was substantially different, in terms of NEMA spatial resolution, image quality, and NECR for ^68^Ga and ^90^Y compared to ^18^F. But these differences are compensated by the PET/MR scanner technologies and reconstructions methods.

## Background

The Signa PET/MR has MR-compatible silicon photomultiplier (SiPM) detector technology characterized by a superior light detection as compared to conventional PET technology [1–3]. The advantage of SiPMs versus avalanche photodiodes (APDs) is a faster response, enabling the combination of excellent time-of-flight (TOF) PET (close to 400 ps) imaging with MR scanning. The smaller detector bore and long axial extent (25 cm) of the PET ring (in comparison to state-of-the-art PET/CT) result in a superior sensitivity of 21 cps/kBq, thus allowing a lower PET tracer dosing besides the evident dose reduction by omitting the CT [4]. Hybrid PET/MR is a relatively new multimodality imaging technique and offers the potential for combined structural, functional, and molecular imaging assessment of a wide variety of oncologic, neurologic, cardiovascular, and musculoskeletal conditions [5, 6]. However, the challenges beyond those of a technical nature remain for PET/MR imaging, including the standardization of appropriateness criteria, image acquisition parameters, and clinically relevant and as well commercially available isotopes.

With PET becoming more widely used, the transport logistics have allowed faster shipments of radioisotopes to small imaging centers. The majority of PET studies in clinical routine are still being performed with ^18^F, because of its physical properties combined with efficient transportation logistics which widely increase its availability. The same holds for ^68^Ga and ^90^Y, of which the use is not dependent on the availability of a cyclotron. However, the physical properties of the PET radioisotopes are quite different from ^18^F. ^18^F almost exclusively decays via positron emission (96.8%) and with a relatively low maximum energy of the positron of 0.6335 MeV. The maximum and mean range of ^18^F are equal to 2.4 and 0.6 mm. The other 3% of decays is via electron capture [7].

The use of the generator-based isotope ^68^Ga has seen a steady increase in the last years. It is obtained from a ^68^Ge/^68^Ga generator obviating the need for a cyclotron on site. One generator will typically be used for about 1 year [8] and the equilibrium between ^68^Ga and ^68^Ge is re-attained rapidly enough to allow multiple radiotracers preparations a day. ^68^Ga is used for labeling both small compound and macromolecules, such as ^68^Ga-PSMA targeting the prostate-specific membrane antigen or ^68^Ga-labeled tracers targeting the somatostatin receptor expressed by neuroendocrine tumors, which are considered as key applications for combined PET/MR [9–11]. ^68^Ga is not a pure positron emitter and has a more complex decay scheme than ^18^F. Non-pure isotopes emit additional gammas that may even directly fall into the energy window accepted by the PET scanner. These high-energy gammas have some probability of generating spurious coincidences after scattering in the patient or via *e*^+^/*e*^−^ pair production in the detector or the patient [12–14]. In 87.8% of the decays, ^68^Ga will emit a positron with a maximum energy of 1.899 MeV and a mean energy of 0.89 MeV with a half-life *t*_1/2_ = 67.6 min. The much higher energy of the positron emission (compared to ^18^F) leads to an increased maximum and mean range of 8.9 and 2.9 mm. It also emits additional gammas of 578.52 keV (0.034), 805.83 keV (0.094), 1077.35 keV (3.22), 1261.08 keV (0.094), and 1883.16 keV (0.137).

In the same way, ^90^Y has rapidly gained attention as one of the most widely used therapeutic radioisotope in nuclear medicine. ^90^Y is used in radioembolization of liver tumors. Tiny glass or resin beads called microspheres are administered in the hepatic artery and are transported into the blood vessels at the tumor site. The spheres get physically trapped and the radioactive isotope ^90^Y delivers a high dose (via electrons) of radiation to the tumor. Several centers also use their PET system to image the therapeutic isotope ^90^Y. Studies have shown that ^90^Y-DOTA and ^90^Y-DTPA have potential in intra-vascular radionuclide therapy and ^90^Y can simultaneously work as an imaging agent and a therapeutic [15–17]. ^90^Y is mainly a *β*^−^ emitter with a very small branching ratio for positron production. In 0.003186% of the decays, there will be the emission of an *e*^+^/*e*^−^ pair at 1.76 MeV. As the transition energy is 1.76 MeV, it remains 738 keV kinetic energy to be split between the electron and the positron in order to conserve the null momentum. With a half-life of 64.1 h, ^90^Y produces a weak but useable PET signal [7, 18], as illustrated by several clinical and phantom studies.

Furthermore, these isotopes may be of particular interest for PET/MR in prostate cancer, liver studies, and follow-up of radionuclide therapy. Therefore, it is relevant to determine the performance of PET/MR for these clinically relevant and commercially available isotopes in order to ensure correct functionality and optimal image quality. For PET scanners in particular, the National Electrical Manufacturers Association (NEMA) has defined a standard to assess the performance of the tomographic system, which is widely accepted by manufacturers [19]. The NEMA NU 2–2007 standard identifies ^18^F as the radionuclide to be used for all tests. Due to factors such as positron range, interference with magnetic field, and non-pure emissions with additional gammas, the results may be different when non-conventional radioisotopes, such as ^68^Ga and ^90^Y [20–22], would be used. The aim of this study is to assess the impact of using different PET isotopes for the NEMA tests performance evaluation of the GE Signa integrated PET/MR. NEMA NU 2–2007 performance measurements for characterizing spatial resolution, sensitivity, image quality, accuracy of attenuation and scatter corrections (IQ), and noise equivalent count rate (NECR) were performed using ^18^F and ^68^Ga. For ^90^Y, all tests except NECR tests were also performed.

## Methods

All phantom experiments were performed on the MP24 version of the GE Signa integrated PET/MR whole-body hybrid system, installed in UZ Leuven, Belgium. The MR component of the hybrid system consists of a 3.0 Tesla static magnetic field, a radiofrequency (RF) transmit body coil, and a gradient coil system which provide a maximum amplitude of 44 mT/m and a maximum slew rate of 200 T/m/s. The PET component is comprised of 5 detector rings, each consisting of 28 detector blocks. Total axial FOV is equal to 25 cm. Table 1 contains a summary of important design and performance parameters.

**Table 1:** Design and PET performance specifications

	PET
Axial FOV	25 cm
Transaxial FOV	60 cm
Photodetector	SiPM
Scintillator	LYSO
Crystal element size	25 × 4.0 × 5.3 mm^3^
Electronics	Integrated in-bore
Time resolution	< 400 ps
Energy window	425–650 keV
Coincidence timing window	4.57 (± 2.29 ns)
NEMA NU 2–2007 PET test^a^	
Sensitivity	22.5 cps/kBq
Peak NECR	212.2 kcps
Activity at Peak NECR	18.1 kBq/ml
Scatter Fraction at Peak NECR	44.1%
Spatial Resolution (Axial)	(5.53–6.95) mm at 1 and 10 cm

^a^GE healthcare acceptance tests [23]

The PET detectors are based on a lutetium-based scintillator (LYSO) readout with MR-compatible silicon photomultiplier technology [3]. Before NEMA testing, a well counter calibration scan was performed with ^18^F in a uniform cylindric phantom. As a recommended calibration, the activity injected was measured using two dose calibrators (Capitec–CRC-55tR) with settings for different isotopes. The following measurements were performed according to the NEMA NU 2–2007 protocol.

### Spatial resolution

A high activity concentration of approximately 200 MBq/ml was used to generate point sources (drop of activity raised in a capillary). In total, more than 500,000 counts were acquired. Both axial and transaxial resolution were measured at two different positions in the axial *z*-direction: in the central position of the FOV and at a position a quarter of the total axial FOV away from the center. The source point position was adjusted until all *x-y-z* values fall between ± 2 mm from the required position. At each of these axial positions, the resolution was measured centrally in the FOV (1 cm horizontal offset relative to the center) as well as 10 cm horizontal offset and 10 cm vertical offset relative to the center. Data was reconstructed with filtered back-projection. The full width at half maximum (FWHM) and full width at tenth of maximum (FWTM) of the point source response function in all three directions were determined by one-dimensional response functions along profiles though the image volume in three orthogonal directions.

### Sensitivity

Sensitivity was tested with the NEMA sensitivity phantom, composed of a line source with 5 different thicknesses of aluminum. The 70-cm-long line source was filled with a volume of approximately 2.3 ml. The activity level was equal to 10.7 MBq and 8.7 MBq at scan start for ^18^F and ^68^Ga. For ^90^Y, the activity level was adjusted to 444.9 MBq, to compensate for the low positron abundance and to keep the scan time acceptable. Using this activity, the total number of counts collected was above 2 million counts. This measurement was done in the center of the FOV and at 10 cm away from the center of the FOV. Data was collected for a period of time to ensure that at least 10.000 trues per slice were collected. The system sensitivity was calculated by fitting the decay-corrected count rate of each acquisition to an exponential and extrapolating the value for a hypothetical acquisition with no aluminum tubes over the source (no attenuation). Axial sensitivity profiles were generated by calculating the sensitivity of each slice for the transaxially centered data acquisitions that used only the smallest aluminum tube.

### Scatter fraction, noise equivalent count rate (NECR)

A 70-cm-long plastic tube line source (3.2 mm in inner diameter) was filled with a calibrated activity of 905 MBq and 871 MBq in a 5.0 ml of solution for ^18^F and ^68^Ga, respectively. The line source was inserted 4.5 cm below the central axis of a 70-cm-long cylindrical polyethylene test phantom. The center of the NEMA scatter phantom was positioned at the FOV center and the data were acquired overnight. After twenty-nine frames of data were extracted from the list mode data, NEMA specifications were used to derive the trues, randoms, scatter, and NECR from the prompts dataset in each frame. The results were plotted as a function of effective activity concentration. In addition, the accuracy of count losses and randoms corrections was determined by extrapolating image results from low count rates.

### Image quality, accuracy of attenuation, and scatter corrections

Image quality was measured by acquiring the NEMA image quality phantom. A 5-cm-diameter cylindrical insert filled with Styrofoam pellets was positioned in the center of the phantom to simulate lung tissue. The warm background volume of the phantom was filled with an activity concentration of 5.3 kBq/ml. Four hot spheres with diameters of 10, 13, 17, and 22 mm were filled with an activity concentration 4 times the background for ^18^F and ^68^Ga. For ^90^Y, a higher 8:1 ratio was used since typical contrasts in liver therapies are normally higher than 4:1. The two cold spheres with diameters of 28 and 37 mm were filled with water (except for the ^90^Y image quality test, in which all of the spheres were filled with an 8:1 ratio of activity).

Background activity from outside the scanner FOV was generated by a line source inserted into the same cylindrical phantom as used in the scatter fraction, count losses, and randoms measurement. It contains 116 MBq solution of the isotope used in the measurement and was placed on the bed axially adjacent to the body phantom. The percentage contrast recovery for the hot and cold spheres and the background variability were calculated, as defined in the NEMA standard. The percentage contrast recovery (in an ideal case = 100%) is determined for each hot sphere *j* by

1 $$ {Q}_{S,j}=\frac{\left({C}_{S,j}/{C}_{B,j}\right)-1}{\left({a}_S/{a}_B\right)-1}\bullet 100\left[\%\right] $$

Where *C*_*S*, *j*_ is the average counts of regions of interest (ROIs) on the spheres. These are positioned in the transverse image slice that contains the centers of the spheres. *C*_*B*, *j*_ represents the average counts in the background ROI. The terms *a*_*S*_ and *a*_*B*_ are activity concentration in the hot spheres and background, respectively.

The phantom has also 2 large spheres which are not filled with isotope. For each nonradioactive sphere *j*, the percentage contrast recovery *Q*_*C*, *j*_ was calculated by

2 $$ {Q}_{C,j}=\left(1-\frac{C_{C,j}}{C_{B,j}}\right)\bullet 100\left[\%\right] $$

Where *C*_*C*, *j*_ and *C*_*B*, *j*_ are average counts in the ROI for sphere *j* and average of all background ROI counts for sphere *j.*

In order to determine the percentage background variability *N*_*j*_ as a measure for the image noise for sphere *j* (in an ideal case = 0%), the following equation was used:

3 $$ {N}_j=\left(\frac{SD_j}{C_{B,j}}\right)\bullet 100\left[\%\right] $$

*SD*_*j*_ is the standard deviation of the background ROI counts for sphere *j*.

In addition, the central cylinder of the phantom did not contain any activity, and the relative error was calculated to determine the accuracy of scatter and attenuation correction as follows:

4 $$ \Delta {C}_{\mathrm{lung},i}=\frac{C_{\mathrm{lung},i}}{C_{B,i}}\bullet 100\left[\%\right] $$

Where Δ*C*_lung, *i*_ is the relative error per percentage units for each slice *i*, *C*_lung, *i*_ is the average counts in the lung insert ROI, and *C*_*B*, *i*_ is the average of the 60 (37 mm) background ROIs drawn for the image quality analysis [19].

### Image-quality phantom images

The phantom data were obtained with a 10-min scan for ^18^F and ^68^Ga acquisitions. For ^90^Y, a long 15-h scan time and a shorter 30 min representing a clinical acquisition were obtained using list mode data selection. The image quality phantom was reconstructed in a volume of 89 images using ordered subset expectation maximization reconstruction algorithm (OSEM) including time-of-flight (TOF) and scatter corrections with 2, 3, and 4 iterations of 28 subsets. The scatter correction for ^68^Ga and ^90^Y were defined as dirty emitters to account for the gamma. These isotopes allow an additional fitting parameter in the scatter tail scaling process [22]. All reconstruction schemes were performed using a matrix size of 256 × 256 with 2.08 × 2.08 × 2.78-mm^3^ voxel size, with 2 mm and no post-smoothing with and without point spread function (PSF). The GE PET/MR uses a system-generated approach that includes a CT-based template attenuation correction for the NEMA IQ phantom.

## Results

### Spatial resolution

The spatial resolution (FBP reconstruction) results for each isotope are presented in Table 2. As a double check, the ^18^F-measured values (at the same equipment across all isotopes) were used as a reference for all measurements performed on the GE Signa PET/MR. The FWHM radial and tangential resolution is slightly degraded for ^68^Ga and ^90^Y. In comparison with ^18^F-measured values, the relative differences were 17.8% and − 1.3% at 1 cm and 27.9% and 3.5% at 10 cm in the axial direction for ^68^Ga and ^90^Y, respectively. With regards to the FWTM in the axial resolution at 1 and 10 cm off-center, the percentage difference relative to ^18^F values were 70%, and 3.3% at 1 cm and 57.3% and 12.3% at 10 cm off-center for ^68^Ga and ^90^Y, respectively.

**Table 2:** Spatial resolution tests

	^18^F	^68^Ga	^90^Y
FWHM (mm)			
At 1 cm			
Transverse^a^	4.05	4.18	4.42
Axial	6.08	7.16	6.00
At 10 cm			
Radial	5.75	5.80	5.79
Tangential	4.38	4.6	4.44
Axial	6.85	8.76	7.09
FWTM (mm)			
At 1 cm			
Transverse^a^	8.62	8.68	9.02
Axial	11.97	20.35	12.36
At 10 cm			
Radial	10.68	10.70	10.78
Tangential	8.61	8.78	8.84
Axial	14.01	22.04	15.73

^a^Transverse (radial and tangential values are averaged together)

### Sensitivity

Sensitivity test results for ^18^F, ^68^Ga, and ^90^Y are 21.8, 20.1, and 0.653·10^−3^ cps/kBq at the center position and 21.2, 19.7, and 0.667·10^−3^ cps/kBq at 10 cm off-center. Table 3 gives the measured average sensitivity values at the transaxial center and 10 cm off-center. Also, data are compared to the average ^18^F measured and theoretical values. These estimated values were calculated based on the difference in branching ratio relative to the ^18^F. The sensitivity values are in line with the lower positron fraction of the isotopes.

**Table 3:** Sensitivity test results: comparison between the average sensitivity measured and theoretical values relative to ^18^F

Isotope	Branching ratio	Average sensitivity measured (cps/kBq)	Theoretical values (cps/kBq)^a^
^18^F	0.968	21.5	–
^68^Ga	0.879	19.9	19.5
^90^Y	31.86·10^−6^	0.6 6 · 10^−3^	0.71·10^−3^

^a^Values relative to ^18^F-measured value

### Scatter fraction, noise equivalent count rate (NECR)

**Fig. 1:**
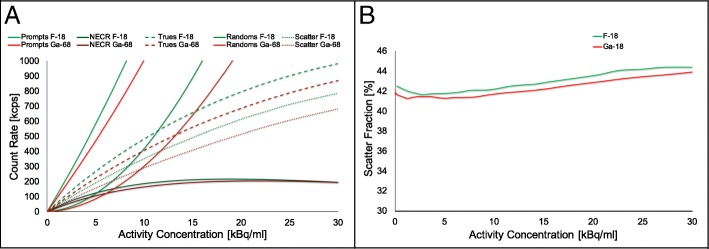
NEMA counting rate measurements: count rates (**a**) and scatter fraction (**b**) vs activity concentration for the both isotopes ^18^F and ^68^Ga. Notice than peak of the NECR curve (**a**) of ^68^Ga is lower (at clinical NECR) and appears at higher activity concentrations

The peak NECR, the corresponding activity concentration and scatter fraction at peak NECR, is presented in Table 4 and Fig. 1 for ^18^F and ^68^Ga. Table 4 summarizes the comparison between ^18^F and ^68^Ga in terms of scatter fraction at peak NECR, peak NECR, activity concentration at peak NECR, and maximum absolute error.

**Table 4:** Scatter Fraction, peak NECR, and source activity test results for ^18^F and ^68^Ga

Scan type	Unit	Measured values	
Isotopes		^18^F	^68^Ga
Scatter fraction at peak NECR	%	43.3	42.9
Peak NECR	kcps	216.8	205.6
Activity at peak NECR	kBq/ml	18.60	20.40
Maximum absolute error	%	3.0	6.0

For ^18^F, the NECR has a maximum of 216.8 kcps at an activity concentration of 18.6 kBq/ml. At the peak, the scatter fraction was 43.3%, comparable to the results obtained on three separate scanners installed in three institutions [23].

For ^68^Ga, the trues, random, and scatter count rates at the same activity concentration are lower in comparison to ^18^F. The measured NECR peak for ^68^Ga was also clearly lower and peaks at 205.6 kcps. This peak is obtained at a higher activity concentration of 20.4 kBq/ml. The scatter fraction at peak NECR was below 1% lower compared to ^18^F measured in our institution (Fig. 1a and Table 4). After the full 15-h acquisitions (for both isotopes), the maximum absolute value of the slice error was 3.0% and 6.0% for ^18^F and ^68^Ga.

### Image quality, accuracy of attenuation, and scatter corrections

**Fig. 2:**
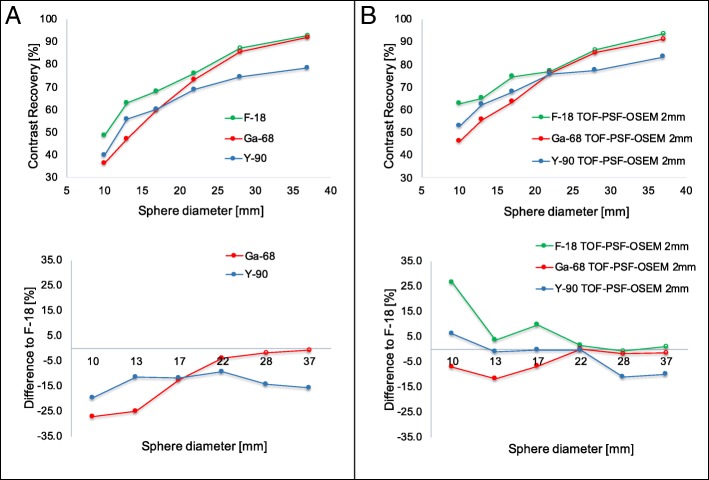
Contrast recovery of TOF-OSEM 4 iteration and 28 subsets as a function of sphere size for 10 min (^18^F and ^68^Ga) and 15 h (^90^Y) acquisition times and different isotopes. The cold and radioactive spheres are indicate using open (^18^F and ^68^Ga) and filled circles (^90^Y), respectively. Contrast recovery and percentage difference relative to ^18^F for each sphere size: **a** without PSF and post-smoothing filter and **b** with PSF and 2 mm post-smoothing filter

The results for contrast recovery versus sphere diameter of the image quality phantom are shown in Fig. 2. In Fig. 2 a, the contrast recovery of the reconstructed image of the phantom without PSF and post-smooth filter was lower for ^68^Ga and ^90^Y as shown in the relative difference to ^18^F-measured values. The contrast recovery increased when compared using TOF, PSF, and post-smooth filter, as showed in Fig. 2 b.

**Fig. 3:**
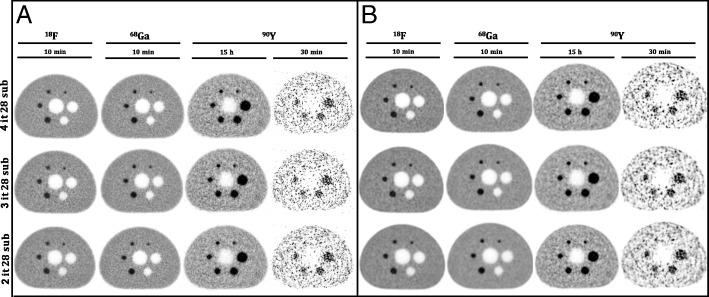
Axial slices of the phantom reconstructed using TOF-OSEM for 2, 3, and 4 iterations and 28 subsets for different acquisition times and different isotopes: **a** without PSF and post-smoothing filter and **b** with PSF and 2 mm Gaussian filter

The background variability and the lung region relative error for ^18^F, ^68^Ga, and ^90^Y are shown in Table 5 for different sphere sizes. ^90^Y has a much lower quality and suffers from low counts, for most spheres the background variability. The lung error is clearly higher than for ^18^F and ^68^Ga. This is also visually confirmed by the clinical reconstructions (30 min acquisition) in Fig. 3.

**Table 5:** Background variability and lung error of TOF-OSEM 4 iteration and 28 subsets image reconstructed, with and without PSF and 2 mm post-smooth filter for ^18^F, ^68^Ga, and ^90^Y respectively

	TOF-OSEM^a^			TOF-PSF-OSEM 2 mm		
	^18^F	^68^Ga	^90^Y	^18^F	^68^Ga	^90^Y
Background variability [%]						
10 mm	6.1	7.5	9.0	7.4	7.2	8.6
13 mm	5.0	5.8	7.9	6.2	6.0	7.7
17 mm	4.2	4.4	7.0	5.0	4.7	6.9
22 mm	3.3	3.3	6.3	4.0	3.5	6.2
28 mm	3.1	2.7	5.9	3.6	2.6	5.7
37 mm	2.7	2.1	4.9	3.0	2.0	4.7
Lung error [%]	1.6	1.1	6.4	1.4	1.0	4.3

^a^4 iterations and 28 subsets

When compared in terms of number of iterations (Fig. 3), the noise level increased with increasing of the number of iterations for all isotopes.

## Discussion

The aim of this study was to assess the impact of using different PET isotopes for the NEMA tests performance evaluation of the GE Signa integrated PET/MR. The performance and characteristics of PET/MR have been investigated based on NEMA NU 2–2012 with regard to ^18^F [20, 21, 23], but not for different PET isotopes. Furthermore, the NEMA NU 22012 version is only slightly different from the 2007 version; the most substantial changes are relatively minor, mostly designed to make the test easier to conduct, mere reproducible, or more clearly defined [19]. In this study, we conveniently choose to evaluate the PET/MR using NEMA NU 2–2007 because GE Healthcare has reported their acceptance testing on this version [24].

The system performance of the GE Signa integrated PET/MR was substantially different, in terms of NEMA spatial resolution and image quality for ^68^Ga and ^90^Y PET imaging test as compared to ^18^F. In the transverse plane, the magnetic field reduces the effective positron range, and the dominant factor on spatial resolution seems to be the detector pixel size and the transverse resolution is therefore comparable with the result of ^18^F. This effect was confirmed by other studies with simulations for different isotopes and field strengths [25, 26]. The main magnetic field along the axial direction leads to an increased positron range in this direction and a pronounced reduction of the range in the transversal plane for high-energy positrons. On the other hand, the positron range effect of the magnetic field is not significant for ^18^F [27] and no significative effect was found for ^90^Y. In agreement with these studies, the FWHM difference relative to ^18^F-measured values were 17.8% and − 1.3% at 1 cm and 27.9% and 3.5% at 10 cm off-center in the axial direction for ^68^Ga and ^90^Y, respectively, as shown in Table 2. However, the NEMA spatial resolution test is designed to characterize the detector, rather than the isotope which leads to limitations to account the effect of magnetic field on positron range on the transaxial resolution measurements. The capillary is very small, and any positron that escapes the capillary is not accounted for in the measurements. In addition, axially, the annihilation could occur in the tube-sealing compound and beyond that, the axial test is slightly poor due to the rebinning process and larger pixel size in the *z*-axis.

The NEMA image quality test was also substantially different in the measured contrast recovery, as shown in Fig. 2. It seems that the inferior resolution also affects the contrast recovery of the radioactive spheres in the NEMA quality phantom for ^68^Ga and ^90^Y. While the results look visually (Fig. 3) similar between ^18^F and ^68^Ga for TOF-OSEM without resolution modeling and post-smooth filter, there is (Fig. 2a) a clearly lower contrast recovery for the smaller spheres in ^68^Ga and also lower contrast recovery in ^90^Y, which is probably caused by the increased positron range and loss in resolution. A similar approach using different PET isotopes in a brain phantom measured at different field strengths was conducted by Shah et al. [27]. The contrast of the reconstructed image of the brain phantom filled with ^68^Ga was significantly affected by the magnetic field in the axial direction more than ^18^F (low-energy positron emitter). However, errors in scatter correction and the use of different sphere to background ratios might have influenced these results [3, 23, 28, 29]. A low-frequency offset of the data makes the images appear to have more or less contrast recovery. And different ratios lead to different contrast recovery. This can explain the crossing of the ^90^Y curve (ratio 8:1) in Fig. 2.

With regards to noise level and the average lung residual error (Table 5), the results were comparable between ^18^F and ^68^Ga, but for ^90^Y, the background variability and the lung error are clearly higher than for ^18^F and ^68^Ga, which is also visually seen (15 h and 30 min acquisition) in Fig. 3. However, when comparing the reconstructed images using resolution modeling and 2 mm post-smooth Gaussian filter to reduce the noise, the contrast recovery increased with acceptable noise level, as shown in Figs. 2b and 3b. Although high-energy positron emitters are affected by the field strengths, recent developments in reconstruction methods including dedicated positron range correction have successfully corrected this effect [30]. A new Bayesian penalized likelihood reconstruction algorithm which uses a block sequential regularized expectation maximization as an optimizer (including TOF and PSF) was introduced in the last few years by GE Healthcare (Q.Clear) on their PET scanners in order to improve clinical image quality. Unlike traditional OSEM reconstruction, which increases the noise with the number of iterations (Fig. 3), this algorithm improves image quality by controlling noise amplification during image reconstruction [31].

The mean sensitivity results shown in Table 3 are in line with theoretical values as expected for ^68^Ga and ^90^Y test. The primary factor for the sensitivity change is the scale factor related to the positron emission fraction (96.7%, 87.9%, and 0.003186% for ^18^F, ^68^Ga, and ^90^Y, respectively). The low branching ratio of ^90^Y explains the substantial quality difference of the reconstructed transverse image quality phantom as compared with ^18^F and ^68^Ga images, as shown in Fig. 3. In several design factors (Table 2) including Compton scatter recovery [31, 32], the longer axial FOV and reduced detector ring diameter lead to higher count rates and an increased sensitivity, both in stand-alone operation and with simultaneous MR image acquisition [33].

NEMA count rate performance and accuracy measurements summarized in Table 4 and Fig. 1a suggest that the scanner provides excellent accurate quantitative measurements and utilizes effective randoms and dead time correction methods for ^18^F [23]. For ^68^Ga, the scatter fraction at NECR peak (Table 4 and Fig. 1b) was slightly lower compared to ^18^F measured in our institution and the GE test. This is primarily due to 1.2% (1.883 MeV) fraction of ^68^Ga that decays by ${\beta}_2^{+}$ can result in a small prompt gamma (1.077 MeV) [12–14] contamination into the PET data. The prompt gammas of ^68^Ga can directly fall into the energy window and accepted by the PET scanner. This happens when the 1.077 MeV scatters in the phantom and generates an energy falling in the main energy window. In this case, there will be a coincidence with a true 511 keV resulting from the same decay [18]. Contributions in which only the gamma is detected would add to randoms which does not affect the calculation for scatter fraction.

The ^68^Ga NECR test was clearly lower than measured for ^18^F and appears at a slightly higher activity concentration (Fig. 1a). The lower peak NECR can be explained by the additional 1.077 MeV gamma which leads to additional detections increasing the deadtime of the detector blocks. These can also lead to additional randoms or scatter when they lose enough energy for falling into the energy window. However, the effect from these prompt-gammas is clearly very small from a scatter fraction perspective and concerning for all activity concentration values below NECR (Table 4), and the maximum absolute value of the slice error is 2.9% and 6.0% for ^18^F and ^68^Ga, respectively. There is also no appreciable impact in the measured residual activity of the lung insert in the IQ phantom (Table 5).

In summary, the overall GE Signa PET/MR system performance with TOF capability based on SiPM detectors, shows substantially different system characteristics for each of these commercially available isotopes. However, the NEMA spatial resolution test is designed to characterize the detector, rather than the isotope, which needs to be adapted in order to well account for the effect of magnetic field on positron range on the transaxial resolution measurements. The variety of prompt gammas in coincidences of these isotopes and the interference of the MR field on positron range, however, seem to have been compensated by the PET scanner technologies, which, in combination with recently developments in reconstruction methods (regularized TOF OSEM and PSF), lead to a comparable noise equivalent count rate and a good scatter fraction.

## Conclusions

NEMA NU 2–2007 performance measurements using ^18^F, ^68^Ga, and ^90^Y resulted in substantially different system characteristics, specifically in terms of spatial resolution and recovery coefficients of the image quality measurements. NEMA spatial resolution test needs to be adapted in order to correctly account for the difference in positron range in the transaxial resolution measurements. And when NEMA image quality test is compared using TOF-OSEM-PSF and post-smooth gaussian filter, the contrast recovery increased with acceptable noise level. The primary factor for the sensitivity change can be explained by the scale factor related to the positron emission fraction of the isotopes. Scatter fraction and NECR differences between the ^18^F and ^68^Ga are relatively small: for ^68^Ga, the peak NECR is lower and appears at higher activity concentrations. The maximum absolute value of the slice error is 2.9% and 6.0% for ^18^F and ^68^Ga, respectively. These performance results are compensated by the PET scanner technologies and reconstructions methods.

## Data Availability

The datasets used and/or analyzed during the current study are available from the corresponding author on reasonable request.

## Abbreviations

CT: Computed tomography
DTPA: Diethylenetriaminepentaacetic acid
FOV: Field of view
GE: General Electric Company
LBS: Lutetium-based scintillator
MR: Magnetic resonance
NECR: Noise equivalent count rate
NEMA: National Electrical Manufacturers Association
OSEM: Ordered subset expectation maximization
PET: Positron emission tomography
PSF: Point spread function
PSMA: Prostate membrane antigen
RC: Recovery coefficient
ROI: Region of interest
SiPM: Silicon photomultipliers
SUV: Standardized uptake value
TOF: Time of flight
VOI: Volume of interest
